# Rumen Epithelial Communities Share a Core Bacterial Microbiota: A Meta-Analysis of 16S rRNA Gene Illumina MiSeq Sequencing Datasets

**DOI:** 10.3389/fmicb.2021.625400

**Published:** 2021-03-15

**Authors:** Chiron J. Anderson, Lucas R. Koester, Stephan Schmitz-Esser

**Affiliations:** ^1^Department of Animal Science, Iowa State University, Ames, IA, United States; ^2^Interdepartmental Microbiology Graduate Program, Iowa State University, Ames, IA, United States; ^3^Department of Veterinary Microbiology and Preventive Medicine, Iowa State University, Ames, IA, United States

**Keywords:** rumen wall, rumen epithelium, epimural, microbiota, ruminant

## Abstract

In this meta-analysis, 17 rumen epithelial 16S rRNA gene Illumina MiSeq amplicon sequencing data sets were analyzed to identify a core rumen epithelial microbiota and core rumen epithelial OTUs shared between the different studies included. Sequences were quality-filtered and screened for chimeric sequences before performing closed-reference 97% OTU clustering, and *de novo* 97% OTU clustering. Closed-reference OTU clustering identified the core rumen epithelial OTUs, defined as any OTU present in ≥ 80% of the samples, while the *de novo* data was randomly subsampled to 10,000 reads per sample to generate phylum- and genus-level distributions and beta diversity metrics. 57 core rumen epithelial OTUs were identified including metabolically important taxa such as *Ruminococcus, Butyrivibrio*, and other *Lachnospiraceae*, as well as sulfate-reducing bacteria *Desulfobulbus* and *Desulfovibrio*. Two *Betaproteobacteria* OTUs (*Neisseriaceae* and *Burkholderiaceae*) were core rumen epithelial OTUs, in contrast to rumen content where previous literature indicates they are rarely found. Two core OTUs were identified as the methanogenic archaea *Methanobrevibacter* and *Methanomethylophilaceae*. These core OTUs are consistently present across the many variables between studies which include different host species, geographic region, diet, age, farm management practice, time of year, hypervariable region sequenced, and more. When considering only cattle samples, the number of core rumen epithelial OTUs expands to 147, highlighting the increased similarity within host species despite geographical location and other variables. *De novo* OTU clustering revealed highly similar rumen epithelial communities, predominated by *Firmicutes*, *Bacteroidetes*, and *Proteobacteria* at the phylum level which comprised 79.7% of subsampled sequences. The 15 most abundant genera represented an average of 54.5% of sequences in each individual study. These abundant taxa broadly overlap with the core rumen epithelial OTUs, with the exception of *Prevotellaceae* which were abundant, but not identified within the core OTUs. Our results describe the core and abundant bacteria found in the rumen epithelial environment and will serve as a basis to better understand the composition and function of rumen epithelial communities.

## Introduction

Domesticated ruminants such as cattle (*Bos taurus*), sheep (*Ovis aries*), goats (*Capra aegagrus*), and yaks (*Bos grunniens*) form an important segment of agriculture around the world with over 3.5 billion domesticated ruminants providing a source of high-quality animal protein in the form of meat and dairy products (Cammack et al., 2018). Non-domesticated ruminants also fill essential ecological niches as primary consumers. Ruminants are distinct from monogastric animals in their ability to degrade cellulose and hemicellulose during feed digestion in the rumen, the first of four digestive chambers in the ruminant gastrointestinal tract system (Paz et al., 2018). Digestion of plant material in the rumen is primarily conducted by a complex and diverse community of bacteria, fungi, protozoa, and archaea, and understanding these communities may provide insight into the metabolic processes essential for host animal health, resilience under stress conditions, and feed efficiency.

The rumen microbiota has been classified into three major groups: microorganisms attached to the solid plant material fraction of the rumen contents, microorganisms free-floating in the liquid fraction of the rumen contents, and microorganisms attached to the rumen wall, which is also called rumen epithelium. The rumen wall bacteria are also referred to as epimural bacteria (Mead and Jones, 1981). Many studies have contributed to identifying the solid and liquid fractions of the rumen content microbiota, as well as functional characterization of rumen content microbiomes (Henderson et al., 2015; Mizrahi and Jami, 2018). However, substantially fewer studies have targeted the rumen epithelial microbiota. It has been known for decades that almost the entire rumen epithelium is covered by microorganisms (McCowan et al., 1978, 1980; Cheng et al., 1979; Wallace et al., 1979; Dinsdale et al., 1980; Mead and Jones, 1981; Hill, 1982; Mueller et al., 1984; Rieu et al., 1990). Previous work suggests that these communities differ in composition from the solid and liquid associated microbial communities of the rumen content (Wallace et al., 1979; Sadet et al., 2007; Mao et al., 2015; Holman and Gzyl, 2019). A common concept in microbial ecology is the core microbiome or microbiota, generally defined as OTUs or taxa which are present in the majority of samples from a given environment. These core microbiota taxa are hypothesized to fill important niches and functions within the microbiome (Shade and Handelsman, 2012; Astudillo-García et al., 2017). Previous analyses based on rumen content samples from cattle (Henderson et al., 2015; Holman and Gzyl, 2019) have contributed to establishing a core rumen content microbiota. This study provides the first step to identify the core rumen epithelial microbiota.

A number of studies have been conducted over the last 10 years to investigate the composition of the rumen epithelial microbiota (Sadet-Bourgeteau et al., 2010; Chen et al., 2011; Li M. et al., 2012; Li R. W. et al., 2012; Petri et al., 2013; Liu et al., 2015, 2016; Wetzels et al., 2015; McCann et al., 2016; De Mulder et al., 2017; Schären et al., 2017; Sbardellati et al., 2020) leading to a considerable increase of our knowledge about the composition of rumen epithelial microbial communities in different ruminants. However, the knowledge about functional properties of rumen epithelial microbial communities is still highly limited, with a few notable exceptions (Wallace et al., 1979; Jin et al., 2016; Mann et al., 2018). Because rumen epithelial microorganisms are directly associated with host tissue, it is likely that they play an integral role in ruminant metabolism and host-microbe interactions, including nutrient exchange across the rumen epithelium (Cheng et al., 1979).

Nowadays, amplicon sequencing using the Illumina MiSeq sequencing platform is a commonly used method to analyze microbial community composition by amplifying and sequencing selected hypervariable regions of the 16S rRNA gene (Goodrich et al., 2014; Knight et al., 2018). For analysis of amplicon sequencing data from different studies, closed-reference OTU clustering and *de novo* OTU clustering approaches can be used, and each provides distinct advantages and disadvantages. *De novo* OTU clustering allows for identification of novel diversity because it does not reject sequences with low similarity to sequences in the reference database. However, it will not be able to cluster reads across multiple hypervariable regions of the 16S rRNA gene into the same OTU, and is therefore unable to meaningfully compare between studies at the OTU level of specificity across a varied dataset such as the one presented in this analysis (Goodrich et al., 2014) and is primarily used in our study at the phylum and genus levels. Closed-reference OTU clustering can cluster sequences from multiple hypervariable regions of the 16S rRNA gene into the same OTU if each sequence maps to the same full-length reference sequence. This allows for meaningful comparison between OTUs across studies which sequenced different hypervariable regions of the 16S rRNA gene as long as the same reference database is used (Callahan et al., 2017). However, novel sequences may be excluded from closed-reference clustering datasets. This study performed both closed-reference and *de novo* OTU clustering, and provides results from each for different analyses, mitigating the disadvantages while maintaining the advantages of each clustering approach. We aimed to identify a core rumen epithelial microbiota by creating a unified data set of publicly available 16S rRNA gene amplicon sequencing data generated using Illumina MiSeq sequencing technology of rumen epithelial samples from a broad set of studies. In total, 17 datasets that met our inclusion criteria regarding read depth and quality were downloaded from the NCBI SRA and the ENA repositories. Here, we provide the first in-depth meta-analysis of the rumen epithelial bacterial microbiota providing evidence for the presence of abundant rumen epithelial microbial phylotypes independent of the differences between the studies included.

## Materials and Methods

### Data Collection

We performed a literature search for studies with published 16S rRNA gene amplicon sequencing data from the epithelial fraction of the rumen as of November 2019. We identified 17 studies comprising 342 rumen epithelial samples that met our criteria for inclusion (Illumina Miseq sequencing, and a minimum read depth of 10,000 sequences after quality control on a per-sample basis) available through NCBI SRA and the European Nucleotide Archive and downloaded these samples via the NCBI sra-toolkit. A list of all studies and samples included in this analysis is available in Supplementary Table 1.

### 16S rRNA Gene Amplicon Sequence Preprocessing and Quality Control

After data collection, all samples were pooled by file type (assembled contigs or separated paired-end reads). Contigs were stringently quality filtered (Q average of 35 or greater over the full sequence) using mothur v1.43.0 (Schloss et al., 2009). Paired-end reads were merged and quality filtered using the “make.contigs” command in mothur (Schloss et al., 2009). Subsequently, all samples were pooled and filtered using a minimum read length of 250 bp, a zero ambiguities threshold, and a maximum homopolymer length of eight bases. Possible chimeric sequences were removed using the “chimera.vsearch” command in mothur using the SILVA.gold reference database provided by the mothur website. This initial quality filtered data set of 19.3 million high quality sequence reads was aligned against the SILVA SSU database release 132 (Quast et al., 2012) before closed-reference and *de novo* OTU clustering using a 97% similarity threshold in QIIME2 (Bolyen et al., 2019) and mothur (Schloss et al., 2009), respectively.

### Closed-Reference OTU Clustering and Generation of Shared OTU Tables

Closed-reference OTU clustering was performed to enable clustering of sequences deriving from different hypervariable regions of the 16S rRNA gene into the same OTU by clustering to the same full-length 16S rRNA gene sequence in the reference database. Datasets included in this study targeted different hypervariable regions of the 16S rRNA, including V3-V5, V3-V4, V4, and V2-V3, thus necessitating this technique for identifying OTUs shared between datasets. More information about the hypervariable regions used in each dataset is available in Supplementary Table 1. Closed-reference OTU clustering was performed with a 97% similarity threshold to the reference sequences in the SILVA SSU 132 release reference database using QIIME2’s (Bolyen et al., 2019) implementation of VSEARCH (Rognes et al., 2016). OTU information and representative sequences were then exported to mothur for taxonomic classification with the SILVA SSU 132 release. This was done to ensure that the classification process was uniform between the closed-reference and *de novo* OTU clustering-based analyses.

Closed-reference clustered OTU data was exported for analysis with Phyloseq v1.32 (McMurdie and Holmes, 2013) in R 3.6.2 (R Core Team, 2020). Beta diversity between samples was visualized using principle coordinates analysis (PCoA) based on Bray-Curtis distances. Samples were not subsampled to a uniform sequencing depth due to the presence of multiple samples with low read depth after closed-reference clustering (fewer than 5,000 reads).

The percentage of samples in which each OTU was detected was then calculated, resulting in a list of 57 OTUs that were found in ≥ 80% of samples, which we considered to be core rumen epithelial OTUs. An additional 90 OTUs were present in ≥ 80% of cattle samples. The threshold of 80% was chosen to account for the high degree of variability in our dataset, allowing us to capture more biologically relevant signal. A higher threshold such as the 90% threshold used in the meta-analysis in Holman and Gzyl (2019) would result in substantially fewer core rumen epithelial OTUs but might exclude potentially important taxa. Further information is available in Supplementary Tables 2, 3. A heat map was generated for visualization of these percentages using the ggplot2 v3.2.1 package (Wickham, 2016) in R using a color palette from the viridis package v0.5.1 (Garnier, 2018).

### *De novo* OTU Clustering and Generation of Community Diversity and Abundance Metrics

*De novo* OTU clustering, in contrast to closed reference OTU clustering, allows for inclusion of sequences with less than 97% similarity to sequences in the reference database, allowing novel diversity to be maintained within the dataset. This is particularly important when working with samples derived from environments containing taxa without high-quality reference sequences. For this reason, *de novo* OTU clustering was also included in this study to generate beta diversity metrics and other whole community measures. Each sample was randomly subsampled to 10,000 reads after quality control using the “sub.sample” command in mothur to reduce bias toward studies with higher sequencing depths. *De novo* OTUs were clustered at 97% similarity using the mothur v1.43.0 “cluster.split” command. OTUs were taxonomically classified using the SILVA SSU 132 release as a reference in mothur. Representative sequences from the 50 most abundant OTUs were queried with NCBI BLAST against the NCBI rRNA/ITS database. The representative sequences were also compared to our rumen genome collection (RGC) a curated database of publicly available rumen derived genomes from whole genome sequencing of organisms in the Hungate 1000 collection (Seshadri et al., 2018) and high quality metagenome assembled genomes (MAGs) from Svartström et al. (2017) and Stewart et al. (2019) using BLAST + (Camacho et al., 2009). A threshold of ≥ 90% nucleotide identity and E value cutoff of < 10^–3^ was used in both cases. The results of these comparisons are presented in Supplementary Table 4.

OTU data was exported to Phyloseq v1.32 in R v3.6.2. Beta diversity between samples was visualized using PCoA based on Bray-Curtis distances. Bray-Curtis distances were also used to quantitatively compare communities by study and by animal type using permutational multivariate analysis of variance (PERMANOVA) and permutational multivariate analysis of dispersion (PERMDISP2) with the “adonis” and “betadisper” commands of the R package vegan v2.5.6 (Jari Oksanen et al., 2019), the results of which are available in Supplementary Table 5. The tax_glom function of Phyloseq was used to aggregate abundance data by phyla and by genera to generate relative abundance plots of the 10 most abundant phyla and 15 most abundant genera across all samples, which were plotted by averaging abundances by study and by animal. All figures were generated with the ggplot2 package v3.2.1 (Wickham, 2016) using color palettes from the viridis package v0.5.1 (Garnier, 2018).

## Results

### Closed-Reference OTU Clustering

**FIGURE 1:**
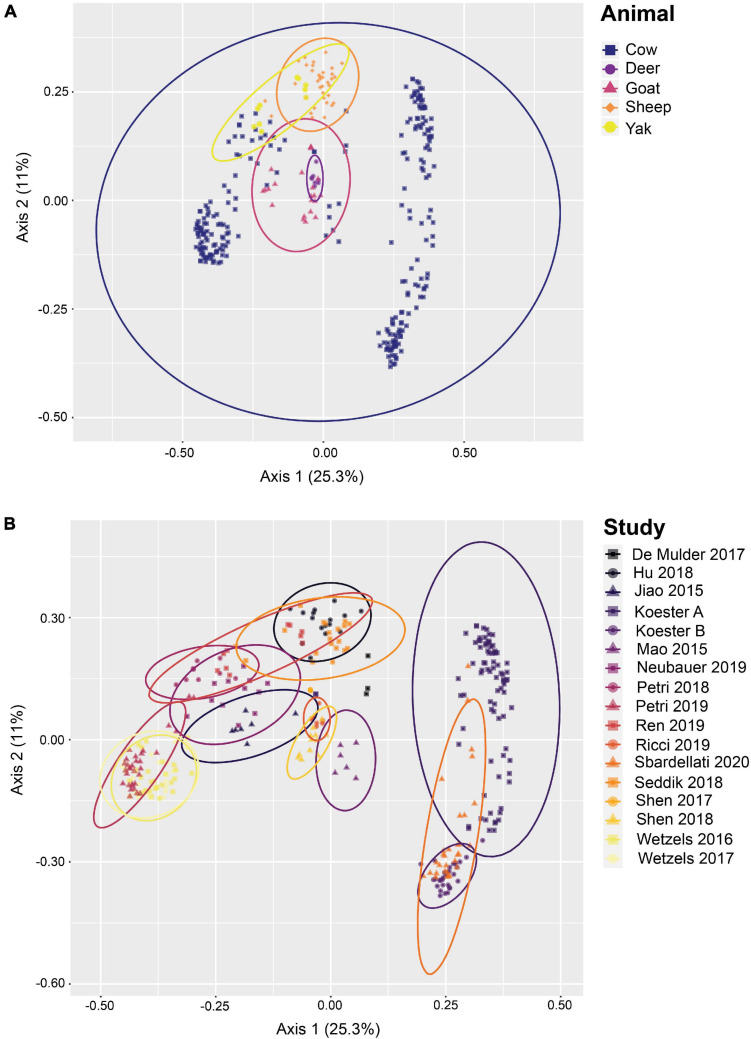
Beta diversity of rumen epithelial microbial communities revealed by principle coordinates analysis based on Bray-Curtis distances after closed-reference OTU clustering. **(A)** Samples highlighted by animal, **(B)** shows samples highlighted by study. See Supplementary Table 1 for more details on the studies.

The 342 samples from 17 different studies contained 19,254,761 high-quality sequences after removal of low quality and potentially chimeric sequences. After closed-reference OTU clustering, 21,850 OTUs representing 14,563,483 sequences were used for downstream analysis. The average number of high-quality sequences remaining per sample was 42,583 sequences, with a standard deviation of 31,732 sequences. 97.7% of all sequence reads were classified as bacterial, 2.1% of the sequence reads as archaeal, and 0.2% as unknown. The 21,508 closed-reference OTUs were classified into 53 phyla, with *Firmicutes*, *Proteobacteria*, and *Bacteroidetes* as the three most abundant phyla at 41.8, 20.0, and 19.6% relative abundance, respectively, across the entire data set. Altogether, these three phyla accounted for 81.4% of all sequences. The most abundant genera were *Campylobacter* (8.4%), *Prevotella* (6.0%), *Christensenellaceae* R-7 group (5.8%), *Butyrivibrio* (5.4%), and an uncultured genus of the *Neisseriaceae* (3.7%). PCoA of all samples (Figure 1) provides evidence of rumen epithelial community clustering by study as well as animal.

**FIGURE 2:**
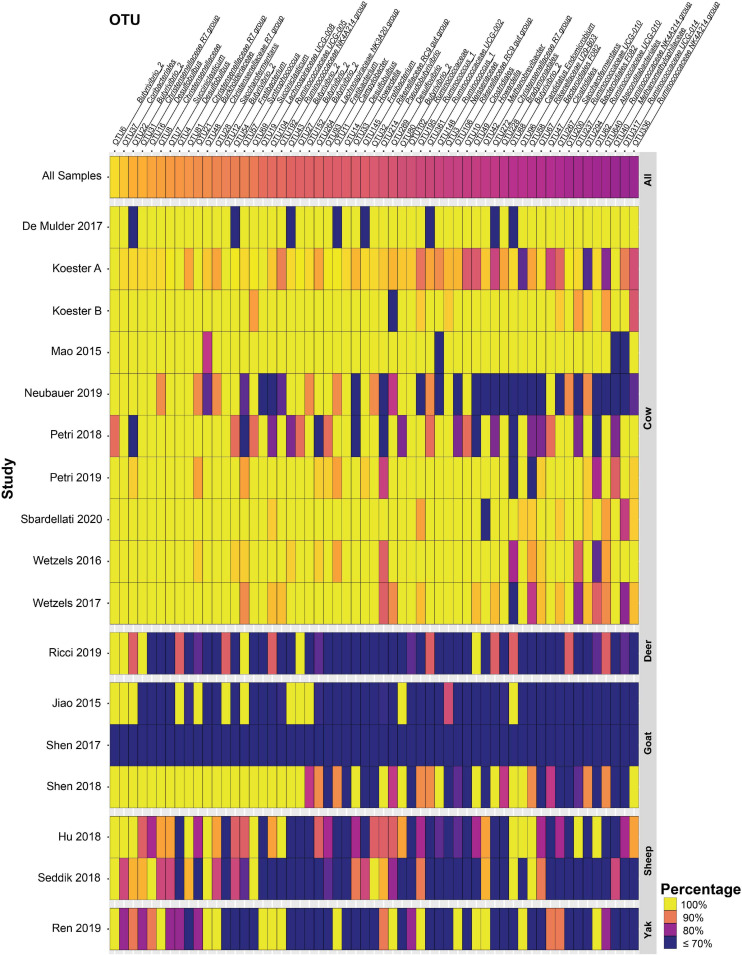
Heat map of 57 core rumen epithelial OTUs (OTUs present in ≥ 80% of samples after closed-reference clustering) showing OTUs shared between different studies. The color represents the percentage of samples in a study that contain the respective OTU. The percentage across all samples is represented at the top, and studies are sorted by animal. Values less than 70% have been truncated to 70% for greater resolution of high values. See Supplementary Table 1 for more details on the studies.

**FIGURE 3:**
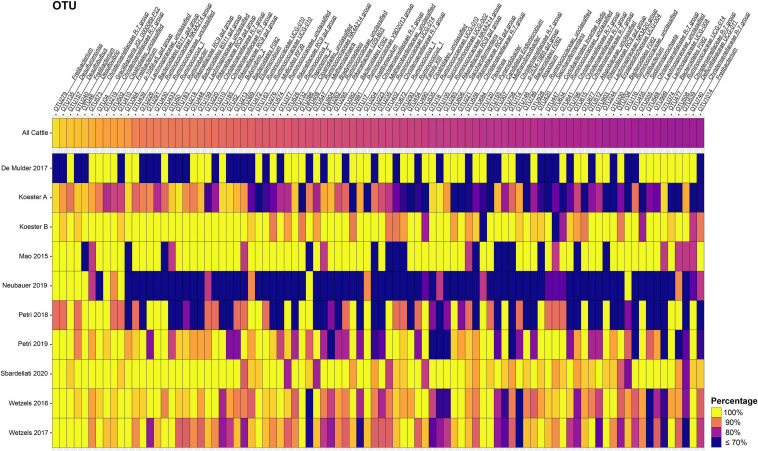
Heat map of the 90 additional core rumen epithelial OTUs identified considering only cattle samples. Only OTUs present in ≥ 80% of cattle samples after closed-reference clustering and not included in the 57 core OTUs shared among all studies presented in Figure 2 are shown. The color represents the percentage of samples in a cattle study that contain the respective OTU. The percentage across all cattle samples is represented at the top. Values less than 70% have been truncated to 70% for greater resolution of the high values. See Supplementary Table 3 for more information about the cattle core rumen epithelial OTUs.

In total, 57 and 16 OTUs were found in 80 or 90% or more of all samples after closed-reference clustering, representing the core rumen epithelial OTUs which are shared between the different studies. These core rumen epithelial OTUs are shown as a heatmap in Figure 2. OTUs 6 and 22 (both classified as *Butyrivibrio*) were present in 98.0 and 94.7% of the samples, respectively. Six other *Butyrivibrio* OTUs (OTUs 12, 152, 27, 43, 80, and 68) were also present in greater the 80% of all samples. Additional core rumen epithelial OTUs in the *Lachnospiraceae* family include *Pseudobutyrivibrio* (OTU 214), *Syntrophococcus* (OTU 57), *Howardella* (OTUs 33 and 49), *Lachnospiraceae* NK3A20 group (OTU 264), and *Lachnospiraceae* UCG-008 group (OTU 19). Other members of the core rumen epithelial OTUs included OTUs classified as *Christensenellaceae* R-7 group (OTUs 31, 48, 81, and 272), *Christensenellaceae unclassified* (OTU 8), and the order *Coriobacteriales* (OTU37). Several core OTUs were identified as *Ruminococcaceae*, including *Ruminococcus* (OTUs 148 and 195), *Ruminococcaceae* NK4A214 group (OTUs 192, 640, and 336), UCG-002 group (OTU 361), UCG-005 group (OTU 104), UCG-010 group (OTUs 200 and 294), and UCG-014 group (OTU 317), *Ruminococcaceae unclassified* (OTU 102), and *Saccharofermentans* (OTUs 28 and 267). Additional core OTUs were identified as *Burkholderiaceae* (OTU 21), *Neisseriaceae* (OTU 3), *Rikenellaceae* RC9 gut group (OTUs 32 and 106), *Desulfobulbus* (OTUs 4, 14, and 16), *Desulfovibrio* (OTU 289), *Succiniclasticum* (OTUs 7 and 69), *Campylobacter* (OTU 11), *Fretibacterium* (OTUs 54, 93, and 145), *Bradymondales* (OTU 228), and *Endomicrobiaceae* (OTU 96). Two core OTUs were identified as archaea, *Methanobrevibacter* (OTU 42) and *Methanomethylophilaceae* (OTU 40). When considering only samples from cattle, a total of 147 OTUs were found in ≥ 80% of samples, and 63 OTUs were present in ≥ 90% of samples (Supplementary Table 3). Of these 147 OTUs, the 90 that were unique to the cattle only core OTUs are presented as a heat map in Figure 3.

### *De novo* OTU Clustering

**FIGURE 4:**
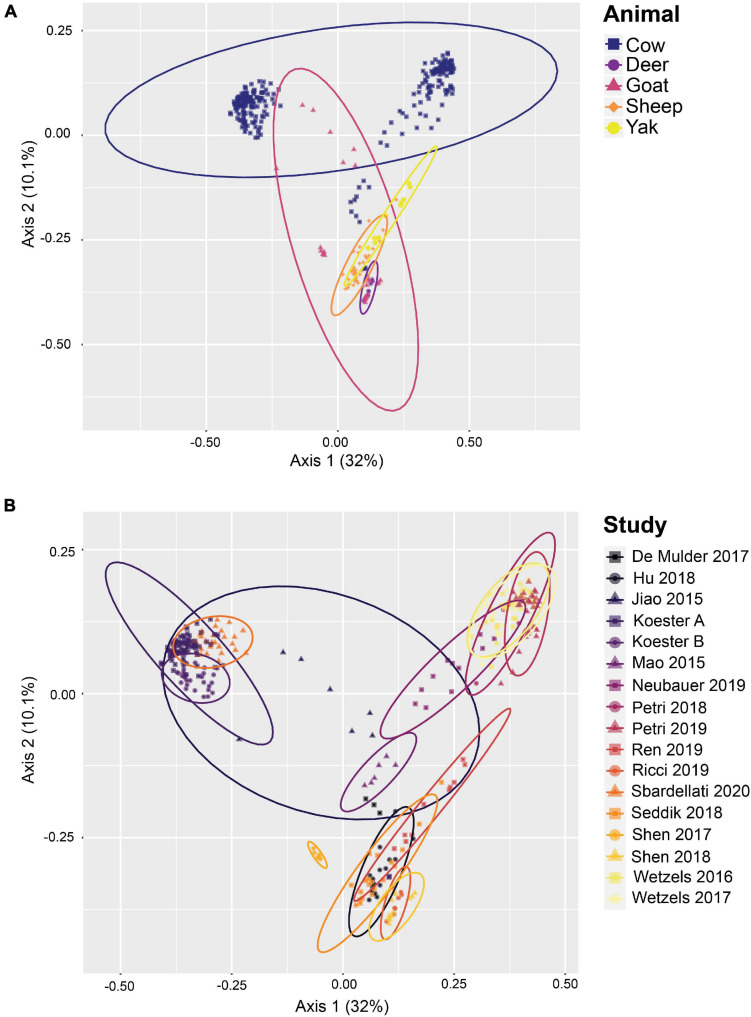
Beta diversity of rumen epithelial microbial communities revealed by principle coordinates analysis based on Bray-Curtis distances after *de novo* OTU clustering. **(A)** Samples highlighted by animal, **(B)** shows samples highlighted by study. See Supplementary Table 1 for more details on the studies.

*De novo* OTU clustering allows the inclusion of novel diversity and is therefore better suited to generating alpha and beta diversity metrics than closed-reference OTU clustering, as well as measuring the relative abundance of taxa across samples from multiple studies. Thus, in addition to closed-reference clustering, *de novo* OTU clustering of the high-quality reads using a 97% similarity threshold was performed after randomly subsampling each sample to a read depth of 10,000, resulting in a total of 71,929 OTUs comprising 3,420,000 reads. 97.4% of all reads were bacterial, 1.9%, of the reads were archaeal, and 0.7% of the reads were classified as unknown. PCoA plots (Figure 4) based on Bray-Curtis distances provided evidence of community clustering by study as well as animal species, as seen after closed-reference OTU clustering. Additionally, the PCoA plots reveal a geographical split between cattle centroids.

**FIGURE 5:**
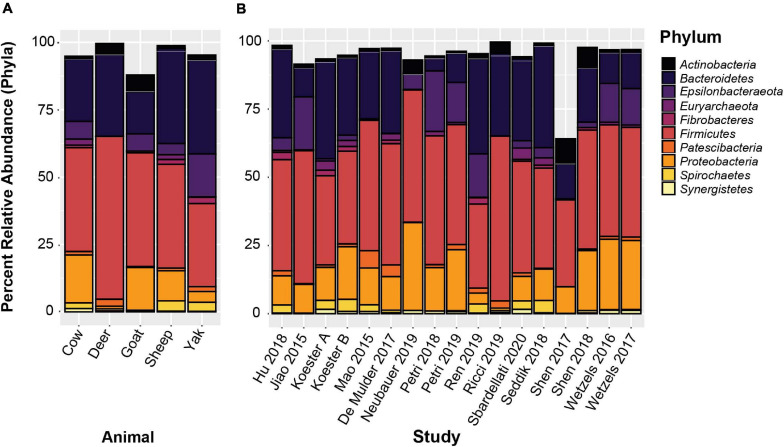
The 10 most abundant phyla based on *de novo* OTU clustering. **(A)** Displays the relative abundances averaged by host animal, **(B)** displays relative abundances averaged by study.

*De novo* clustered OTUs were classified into 53 phyla similar to the closed-reference clustered OTUs. The three most abundant phyla were *Firmicutes*, *Bacteroidetes*, and *Proteobacteria* at 39.1, 24.5, and 16.14% relative abundance across the entire data set, respectively. These three phyla represented 80.3% of all reads, which was highly similar to the results obtained from the closed-reference OTU clustering. Less abundant phyla with a relative abundance greater than 1% were *Epsilonbacteraeota* (6.4%), *Spirochaetes* (2.2%), *Euryarchaeota* (1.9%), *Actinobacteria* (1.4%), *Patescibacteria* (1.2%), *Fibrobacteres* (1.0%), and *Synergistetes* (1.0%). The 10 phyla with the highest relative abundance across all samples are displayed averaged by animal and by study in Figure 5. While variation between relative abundances is present across both animal species and study; overall, the composition of the rumen epithelial microbiota is largely similar, with *Firmicutes*, *Bacteroidetes* and *Proteobacteria* remaining the predominant phyla across most studies. Neubauer et al. (2019), a cattle dataset, had a lower abundance of *Bacteroidetes*, while Ren et al. (2019) and Ricci et al. (2019), the sole yak and deer datasets, respectively, display a lower abundance of *Proteobacteria.*

**FIGURE 6:**
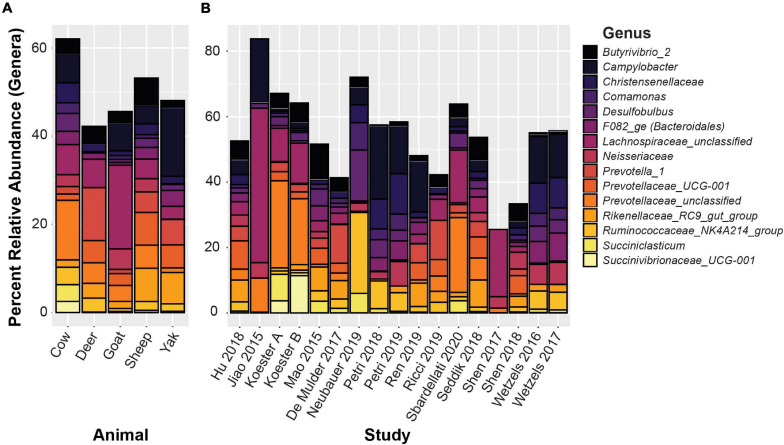
The 15 most abundant genera based on *de novo* OTU clustering. **(A)** Displays relative abundances averaged by host animal, **(B)** displays relative abundance averaged by study.

*De novo* clustered OTUs were classified into 1,297 unique genera. The five most abundant genus-level classifications were *Prevotellaceae* (unclassified) (11.4%), *Lachnospiraceae* (unclassified) (7.3%), *Campylobacter* (6.4%), *Christensenellaceae* R-7 group (3.9%), and *Butyrvibrio_*2 (3.6%). With the exception of unclassified *Lachnospiraceae*, these genera were also highly abundant in the closed-reference OTU clustering. The 15 genera with the highest relative abundance are displayed in Figure 6, representing 59.0% of the total relative abundance across all samples. In addition to the genera mentioned above, these include *Desulfobulbus* (3.4%), *Ruminococcaceae* NK4A214 group (3.4%), *Succiniclasticum* (3.0%), *Bacteroidales* family F082 (2.8%) *Neisseriaceae* (2.7%), *Rikenellaceae* RC9 gut group (2.5%), *Comamonas* (2.0%), and *Succinivibrionaceae* UCG-001 (1.9%). Two additional *Prevotella* and *Prevotellaceae* phylotypes were also present, and the total relative abundance of *Prevotellaceae* within the top 15 genera was 16.0%. The 50 most abundant OTUs with taxonomic information from NCBI Blast and the RGC appended are displayed in Supplementary Table 4. Not surprisingly, more variability was found in the genus-level data compared to the phylum-level analyses. Nevertheless, these 15 most abundant genera represented between 25.5 and 83.8% of all sequences in each of the studies.

## Discussion

### Core Rumen Epithelial OTUs Revealed by Closed-Reference OTU Clustering

Here we perform the first large-scale meta-analysis of rumen epithelial bacterial communities. Our results provide deep insights into common and abundant bacterial phylotypes of the rumen epithelium that are commonly found in spite of differences between individual studies. Closed-reference OTU clustering enabled the creation of a set of 57 core rumen epithelial OTUs found in ≥ 80% of all 342 samples. Given the broad range of host species, diets, farm management practices, geographical locations, hypervariable regions sequenced, and other variables between studies, these core OTUs can be inferred to be remarkably consistent members of the rumen epithelial microbiota. Sixteen core rumen epithelial OTUs were present in ≥ 90% of samples, the threshold used in Holman and Gzyl (2019). Additional information can be found in Supplementary Table 2. When considering only cattle samples a total of 147 OTUs are present in ≥ 80% of samples and 63 OTUs are present in ≥ 90% of samples (Figure 3 and Supplementary Table 3), which suggests that the core rumen epithelial microbiome is larger when considering an individual host species. The previous meta-analysis of the bovine gastrointestinal tract microbiota presented in Holman and Gzyl (2019) likewise found that certain taxa were consistently found across multiple studies and different GI tract locations. Several of the groups identified as core rumen epithelial OTUs have also been identified in their previous meta-analysis of rumen contents. The analysis by Holman and Gzyl (2019) identified four bacterial genera present in ≥ 90% of all 2,662 rumen content samples included in their study, including *Prevotella*, *Ruminococcus*, *Lachnospiraceae* UCG-008, and *Eubacterium coprostanoligenes* group. Several *Lachnospiraceae* core rumen epithelial OTUs were identified in our study, including one *Lachnospiraceae* UCG-008 OTU shared by 84.9% of samples. We also identified several *Ruminococcaceae* core OTUs and two *Ruminococcus* OTUs shared by 81.8 and 80.7% of samples. No core OTUs were identified as either *Prevotella* or *Eubacterium coprostanoligenes*. This suggests that, while there are differences in the core rumen content and core rumen epithelial microbiota, there is also overlap between them.

Eight core rumen epithelial OTUs, including the two OTUs found in the highest number of samples (OTUs 6 and 22), were classified as *Butyrivibrio*, a genus of *Lachnospiraceae* known to produce butyrate, which aids the host in forming tight junctions in the epithelial layer (Peng et al., 2009), as well as providing an energy source. *Butyrivibrio* rumen isolates have been shown to encode a large number of carbohydrate-active enzymes and to be able to degrade and grow on complex plant carbohydrates such as hemicellulose, pectins, mannans, and starch, suggesting they play a role in fiber degradation in the rumen environment (Palevich et al., 2019). Several additional core rumen epithelial OTUs were classified to *Lachnospiraceae* including to the genera *Syntrophococcus* (OTU 57), *Howardella* (OTUs 33 and 49), *Pseudobutyrivibrio* (OTU 214), *Lachnospiraceae_*UCG-008 (OTU 19), and *Lachnospiraceae* NK3A20 group (OTU 264). A quarter of all core rumen epithelial OTUs identified in this study were in the family *Lachnospiraceae*, highlighting their ubiquity in the rumen epithelial community. While *Lachnospiraceae* are commonly found in the GI tract of many animals (including humans) (Sorbara et al., 2020), and in many GI tract segments, they are notable as one of few bacterial taxa in which cellulases are commonly found in the rumen (Moraïs and Mizrahi, 2019). Twelve core rumen epithelial OTUs were classified as *Ruminococcaceae*, including OTUs 192, 336, and 640 which was classified as *Ruminococcaceae* NK4A214 group, a phylotype that has been previously identified to be present in 50% of all bovine GI tract samples (Holman and Gzyl, 2019). Functional characterization of this group in the future may aid in a better understanding of its role in ruminant metabolism as core rumen epithelial and core bovine GI tract taxa. Two *Ruminococcaceae* core OTUs (OTUs 148 and 195) were classified as *Ruminococcus*, members of which have exhibited cellulose-degrading capabilities alongside utilization of many other polysaccharide substrates. Some *Ruminococcus* are capable of crystalline cellulose deconstruction and express GH48 family exoglucanases which were identified as the most highly expressed cellulase (Moraïs and Mizrahi, 2019). Five core rumen epithelial OTUs (OTUs 8, 31, 48, 81, and 272) were classified as members of the *Christensenellaceae* family, with OTUs 31, 48, 81, and 272 further classified as *Christensenellaceae* R-7 group. This group was found to be associated with bovine rumen samples more frequently than fecal samples in the previous bovine GI tract meta-analysis (Holman and Gzyl, 2019). The type strain for this group is *Christensenella minuta*, isolated from human fecal samples which demonstrated butyrate and acetate production (Morotomi et al., 2012). One core rumen epithelial OTU (OTU 37) was classified as a member of the *Coriobacteriales*, an order of commensal bacteria with broad saccharolytic capabilities which produce lactic, and acetic acid as well as ethanol and hydrogen (Gupta et al., 2013, 2017).

Three core rumen epithelial OTUs (OTUs 4, 14, and 16) were classified as *Desulfobulbus*, a genus capable of reducing sulfate to hydrogen sulfide, which can cause inflammation in the GI tract. Expression of the *dsr*AB genes responsible for sulfate reduction has been shown for *Desulfobulbus* in a recent metatranscriptome sequencing study of rumen epithelial samples (Mann et al., 2018), suggesting that sulfate-reducing bacteria actively reduce sulfate at the rumen epithelium. The related sulfate-reducer *Desulfovibrio* has been isolated from the rumen of sheep (Howard and Hungate, 1976). Two core rumen epithelial OTUs (OTUs 3 and 21) were classified as taxa of the *Betaproteobacteria*, *Neisseriaceae*, and *Burkholderiaceae*, respectively. Little is known about the role these *Betaproteobacteria* play in the rumen. *Burkholderiales* and unclassified *Betaproteobacteria* have previously been found to have a positive correlation with Isoflavone-enriched feed in Holstein cows (Kasparovska et al., 2016), while *Neisseria* was positively associated with urea supplementation in the absence of urea hydrolysis inhibitors in a simulated rumen fermentation environment (Jin et al., 2016). However, in both cases, the mechanism which drives the association is unknown. The genus *Burkholderia* contains *B. pseudomallei* and *B. mallei* which are zoonotic pathogens that cause meliodosis and glanders/farcy, respectively. Meliodosis has been confirmed in ruminants (Limmathurotsakul et al., 2012). *Neisseriaceae*-like phylotypes were found to be highly abundant and active on the rumen epithelium in a metatranscriptome sequencing study, which revealed high expression of key genes involved in nitrogen metabolism (Mann et al., 2018). One core rumen epithelial OTU (OTU 11) was classified as *Campylobacter*, a genus which can cause infertility and abortion in cattle (Hoffer, 1981). However, it is unclear whether its presence in the rumen can lead to disease, as it is ubiquitously present in the rumen epithelial community. Rumen epithelial *Campylobacter* phylotypes have been shown to display high expression levels of genes in involved in nitrogen metabolism and in oxidative stress response (Mann et al., 2018). Two core rumen epithelial OTUs (OTUs 32 and 106) were classified as *Rikenellaceae* RC9 gut group. Little is known about this group and their role in ruminant metabolism, but two genera of the *Rikenellaceae* family, *Rikenella* and *Alistipes*, are known to produce VFAs, including acetate, propionate, and succinate (Graf, 2014). OTU 228 is a core rumen epithelial OTU identified as a member of the *Bradymonadales*, an order of bacteria about which relatively little is known, save for the fact that they prey on other bacteria and appear to preferentially prey on *Bacteroidetes* and *Proteobacteria* (Mu et al., 2020), two of the most abundant phyla in the rumen epithelial environment. OTU 96 is a core rumen epithelial OTU identified as a member of the *Endomicrobiaceae* family, a clade which has a tight association as intracellular symbionts of flagellate and ciliate protozoa in termite and cockroach gut environments as well as the rumen microbiota. The protozoa are associated with cellulose degradation in termites, but the function of *Endomicrobiaceae* in the rumen is not well characterized (Zheng et al., 2016; Levy and Jami, 2018). OTUs 42 and 40 are archaeal core rumen epithelial OTUs identified as *Methanobrevibacter* and *Methanomethylophilaceae*, respectively. *Methanobrevibacter* is known to contribute to methane production and is both free living and an endosymbiont of protozoa in the rumen (Levy and Jami, 2018). Little is known about the *Methanomethylophilaceae* family at present, but methylotrophic members have been associated with reduced methane emissions from the rumen (Poulsen et al., 2013). The occurrence of an additional 90 cattle-only core rumen epithelial OTUs (Figure 3) highlights the increased similarity between samples derived from specific species as compared to the broader rumen epithelial community surveyed here. The occurrence of a species-specific epithelial core microbiota may have important implications for understanding each host environment, and as additional data becomes available studies focusing on the core rumen epithelial microbiota of a single host species will be highly valuable in addition to broader approaches.

### Average Relative Abundance of Taxa in Rumen Epithelial Communities Revealed by *de novo* OTU Clustering

In addition to establishing a set of core rumen epithelial OTUs, the other goal of this study was to characterize the most abundant taxa observed in rumen epithelial communities across all studies. The data included in this study reflects the available published rumen epithelial 16S rRNA amplicon sequencing datasets in the literature, which are primarily from cattle. Beta diversity of samples using Bray-Curtis distances (displayed as PCoA in Figure 4) suggests that while samples cluster strongly by study, as seen in previous meta-analyses (Henderson et al., 2015; Holman et al., 2017; Holman and Gzyl, 2019), clustering by animal is also a factor. PERMANOVA and PERMADISP2 results testing these variables are presented in Supplementary Table 5. Two distinct centroids are apparent when examining the distribution of cattle samples, which appear separated in large part by geographic difference. The cluster on the left consists of samples from the United States while the cluster on the right consists of samples from Austria, suggesting that geography may be a factor that contributes the composition of rumen epithelial microbial communities (see Supplementary Table 1). Differences that appear to follow geographic patterns may be driven by climate factors, diet, host lineage, and farm management practices which differ between countries and individual farms, as noted in a previous large-scale analysis of microbial communities in rumen content samples (Henderson et al., 2015).

However, the results of our analysis indicate that, at the phylum level, community makeup between cattle, goats, and sheep are highly similar, and dominated by *Bacteroidetes*, *Firmicutes*, and *Proteobacteria*, which together made up 79.7% of subsampled sequence reads from all included studies. *Proteobacteria* were substantially less abundant in yak and deer. However, each were represented here by a single dataset only. *Epsilonbacteraeota* (6.4% of all reads) showed extremely low abundance in deer samples at 0.001% relative abundance (Figure 5). Diversity in community composition between studies increases when observing communities at the genus (or lowest available taxonomic) level (Figure 6). However, the top 15 genera represent 59.0% of all subsampled sequence reads and averaged 54.5% of sequences in each individual study. This is a remarkable level of similarity between rumen epithelial communities across this highly varied dataset, and these abundant taxa overlap substantially with the core rumen epithelial OTUs identified by closed reference clustering. This overlap includes the taxa *Campylobacter*, *Christensenellaceae* R7 group, *Neisseriaceae*, *Butyrivibrio*, *Desulfobulbus*, *Ruminococcaceae* NK4A214 group, *Succiniclasticum*, and *Rikenellaceae* RC9 gut group, as well as an OTU labeled *Lachnospiraceae* unclassified. *Prevotellaceae* are highly abundant in the *de novo* clustered OTUs, but no *Prevotellaceae* OTUs were identified as core OTUs in the analysis of the closed-reference clustered OTUs.

### Limitations and Future Directions

Meta-analyses of livestock microbiota data has been shown to provide valuable insights into common and shared members of microbiomes as highlighted by two recent studies on pigs and cattle (Holman et al., 2017; Holman and Gzyl, 2019). Nevertheless, there are also several limitations to the dataset analyzed in this study which present challenges when attempting to make inferences about differences in community structure between individual datasets. The host animals in each study received different diets, were maintained with different farm management practices, were sampled at different stages of life, and derived from different geographic regions. Differences in sampling technique, location within the rumen, and washing of the rumen epithelia after sampling may also introduce biases between studies. Deer and yak were represented by a single dataset each, lowering the comparability between cattle and less represented ruminants, since study has been shown in previous meta-analyses to be a major contributing factor to variation in animal microbiome data (Holman et al., 2017; Holman and Gzyl, 2019). We chose to exclude studies that used sequencing platforms other than the Illumina MiSeq platform, particularly pyrosequencing studies, to increase the read depth of the samples, improve overall sequence quality and increase homogeneity of read length distribution. However, the variation in read count between studies remained high. After removal of low quality and chimeric sequences, samples ranged from 10,083 reads to 174,668 reads. To avoid biasing our *de novo* OTU clustering toward OTUs from studies with higher read depth, random subsampling to 10,000 reads per sample was employed. In the closed-reference OTU clustering analysis, sequences with less than 97% similarity to reference sequences were lost, exacerbating the range of sequence depths per sample. Sequence loss during closed-reference clustering should also be considered non-random since it is dependent on the reference database used. In addition to variation in sequencing depth, studies varied by hypervariable region sequenced, primers used, and DNA extraction methods. Hypervariable regions differ in their ability to differentiate and classify taxa (Kim et al., 2011; Yang et al., 2016) while primer choice and DNA extraction methods can introduce their own biases. An alternative to OTUs known as Amplicon Sequence Variants (ASVs) uses exact sequences rather than clustering, which can increase comparability between studies compared to OTU clustering which is contextual (Callahan et al., 2017). However, ASVs do not resolve the difficulty of comparing across different hypervariable regions and were not suitable for this meta-analysis.

Nevertheless, in spite of the limitations mentioned above, our dataset reveals the presence of shared and abundant members of the rumen epithelial microbiota among different ruminants and conditions. Therefore, our results provide a high degree of confidence in establishing core rumen bacterial phylotypes that are present in rumen epithelial communities around the world, across varied diets, management practices, and in different ruminants. Many of the core and abundant rumen epithelial phylotypes have also been found to be abundant in pyrosequencing-based rumen epithelial microbiome studies performed in various ruminants as well as studies using cloning approaches (Cho et al., 2006; Sadet-Bourgeteau et al., 2010; Li M. et al., 2012; Petri et al., 2013; Malmuthuge et al., 2014), which were not included in our analysis. The abundance of these core rumen epithelial phylotypes in pyrosequencing data thus provides additional evidence for the abundance and importance of the core rumen epithelial microbiota identified here. Additionally, several members of the core rumen epithelial microbiota are taxa not commonly found in rumen content, such as *Campylobacter*, *Burkholderiaceae*, *Comamonas*, *Desulfobulbus*, and *Neisseriaceae* (Henderson et al., 2015; Holman and Gzyl, 2019). It is thus highly likely that these members of the core rumen epithelial microbiota have key functions at the rumen epithelium or are better suited to the epithelial environment than the fluid and particulate rumen fractions. It is important to note that most of the members of the rumen epithelial core microbiota have yet to be functionally examined in detail. This is particularly important given the critical role of epithelial and mucosal surfaces for maintaining gut integrity (Aschenbach et al., 2019). The availability of metagenome-assembled rumen genomes, sequencing of functional genes, and rumen epithelial metatranscriptome sequencing have provided first functional insights into the rumen epithelial microbiota (Jin et al., 2017; Mann et al., 2018; Stewart et al., 2019). Given their high prevalence, further functional characterization of these core rumen epithelial microbes through genomic, meta-transcriptomic, and culture-based approaches will be a valuable contribution to our understanding of the rumen epithelial environment, ruminant metabolism, and the impact of these microbes on the host.

## Data Availability Statement

The original contributions presented in the study are included in the article/Supplementary Material, further inquiries can be directed to the corresponding author/s.

## Ethics Statement

Ethical review and approval was not required for the animal study because this meta-analysis study used only already available sequencing datasets from different animal trials. No animal trial was performed in this study. Thus, no animal ethics committee approval was required.

## Author Contributions

SS-E and CA conceived and designed the study. CA analyzed the sequencing data. LK provided sequencing datasets. CA, LK, and SS-E wrote the manuscript. All authors have reviewed and approved the final version of the manuscript.

## Conflict of Interest

The authors declare that the research was conducted in the absence of any commercial or financial relationships that could be construed as a potential conflict of interest.
