# Supplementary material for: Rumen Epithelial Communities Share a Core Bacterial Microbiota: A Meta-Analysis of 16S rRNA Gene Illumina MiSeq Sequencing Datasets
Source: Front Microbiol. 2021 Mar 15;12:625400. doi: 10.3389/fmicb.2021.625400 (PMC8005654; doi:10.3389/fmicb.2021.625400)
Supplement: Supplementary file 5 [file Table_5.pdf]

Supplementary Table 5: PERMANOVA and PERMDISP2 comparing microbial communities by study and animal using Bray-Curtis distances

Clustering Method	Variable	Test	P-value
De novo	Study	PERMANOVA	0.001
De novo	Study	PERMDISP2	0.001
De novo	Animal	PERMANOVA	0.001
De novo	Animal	PERMDISP2	0.001
Closed-reference	Study	PERMANOVA	0.001
Closed-reference	Study	PERMDISP2	0.001
Closed-reference	Animal	PERMANOVA	0.001
Closed-reference	Animal	PERMDISP2	0.001

All tests were conducted in R based on Bray-Curtis distances using the "adonis" and "betadisper" commands of the R package vegan v2.5.6. PERMANOVA results indicate significant differences between the centroids of the communities in all 4 cases. PERMDISP2 tests the hypothesis of equal variance and found that variance is not equal between groups, as expected in this highly uneven dataset. As such it is unclear whether the differences between centroids reflect true differences or are caused by differences in variance, but these differences are represented in Figures 2 and 4 (PCoA of closed-reference and de novo OTU clustered communities respectively).
